# Vertical Interface Induced Dielectric Relaxation in Nanocomposite (BaTiO_3_)_1-x_:(Sm_2_O_3_)_x_ Thin Films

**DOI:** 10.1038/srep11335

**Published:** 2015-06-10

**Authors:** Weiwei Li, Wei Zhang, Le Wang, Junxing Gu, Aiping Chen, Run Zhao, Yan Liang, Haizhong Guo, Rujun Tang, Chunchang Wang, Kuijuan Jin, Haiyan Wang, Hao Yang

**Affiliations:** 1 College of Science, Nanjing University of Aeronautics and Astronautics, Nanjing 211106, China; 2 College of Physics, Optoelectronics and Energy & Collaborative Innovation Center of Suzhou Nano Science and Technology, Soochow University, Suzhou 215006, China; 3 Beijing National Laboratory for Condensed Matter Physics and Institute of Physics, Chinese Academy of Science, Beijing 100190, China; 4 Department of Electrical and Computer Engineering, Texas A&M University, College Station, Texas 77843-3128, USA; 5 School of Physics and Materials Science, Anhui University, Hefei 230039, China

## Abstract

Vertical interfaces in vertically aligned nanocomposite thin films have been approved to be an effective method to manipulate functionalities. However, several challenges with regard to the understanding on the physical process underlying the manipulation still remain. In this work, because of the ordered interfaces and large interfacial area, heteroepitaxial (BaTiO_3_)_1-x_:(Sm_2_O_3_)_x_ thin films have been fabricated and used as a model system to investigate the relationship between vertical interfaces and dielectric properties. Due to a relatively large strain generated at the interfaces, vertical interfaces between BaTiO_3_ and Sm_2_O_3_ are revealed to become the sinks to attract oxygen vacancies. The movement of oxygen vacancies is confined at the interfaces and hampered by the misfit dislocations, which contributed to a relaxation behavior in (BaTiO_3_)_1-x_:(Sm_2_O_3_)_x_ thin films. This work represents an approach to further understand that how interfaces influence on dielectric properties in oxide thin films.

The emergence of novel phenomena and functionalities at artificially constructed oxide heterostructures has stimulated intense research activities over the past decade^1^,^2^. Among these studies, oxide interfaces are very attractive because the coexistence and interplay between different degrees of freedom (charge, orbit, spin, and lattice) at interfaces can lead to rich physical phenomena, including two-dimensional electron gas (2DEG), superconductivity, colossal magnetoresistance, and multiferroic behavior^3^,^4^,^5^,^6^,^7^,^8^,^9^,^10^. For instance, Liu *et al.* demonstrated that oxygen vacancies (*V*_O_s) are the dominant origin of the 2DEG at LaAlO_3_/SrTiO_3_ interfaces when the LaAlO_3_ overlayer is amorphous^6^. A novel ferromagnetic state was observed at the interface between antiferromagnet BiFeO_3_ and ferromagnet La_0.7_Sr_0.3_MnO_3_, which is directly attributed to an electronic orbital reconstruction at the interface^11^.

In addition to these conventional lateral interfaces (parallel to substrate surface), vertical interfaces (perpendicular to substrate surface) in vertically aligned nanocomposite thin films have been introduced and used to create or enhance functionalities of oxide thin films^8^,^9^. Compared to lateral interfaces, vertical interfaces possess impressive advantages, such as reduced clamping effect from substrates, larger interfacial area, strain tunability to larger thickness, and easy interface probing etc^12^,^13^,^14^,^15^. Furthermore, such ordered structures allow for precise tuning of mechanical, electronic, and magnetic properties through vertical strain control, as well as interfacial couplings. For example, Moshnyaga *et al.* showed colossal magnetoresistance effect has been enhanced in (La_0.7_Ca_0.3_MnO_3_)_1-x_:(MgO)_x_ thin films through lattice strain^8^. Zheng *et al.* reported that magnetoelectric coupling has been realized in (BaTiO_3_)_0.65_:(CoFe_2_O_4_)_0.35_ thin films by vertical interfaces coulpings^9^. Besides, vertical interfaces induced strain state reversion and leakage current reduction have been achieved in (BiFeO_3_)_0.5_:(Sm_2_O_3_)_0.5_ thin films^12^,^16^. And enhanced low field magnetoresistance has been reported in heteroepitaxial (La_0.7_Sr_0.3_MnO_3_)_0.5_:(ZnO)_0.5_ via tuning the microstructure and vertical interface density^17^. It is clear that oxide interfaces are effective to control functionalities of oxide thin films. Most previous reports have focused on exploring magnetism, ferroelectricity, magnetoelectric coupling, and electric transportation^6^,^8^,^9^,^10^,^11^. However, the question that arises naturally is whether dielectric properties can be manipulated by oxide interfaces. The work presented here suggests an answer in the affirmative.

Relaxation properties have been approved to be critical for the applications (such as transducers, actuators, and sensors etc.) of dielectric materials^18^,^19^,^20^,^21^. It is highly attractive to manipulate relaxation properties through interfaces, which is also helpful to understand the relationship between oxide interfaces and physical properties. It has been showed that *V*_O_s is responsible for dielectric relaxations observed in epitaxial K_0.5_Na_0.5_NbO_3_/La_0.67_Sr_0.33_MnO_3_ and Ba_0.7_Sr_0.3_TiO_3_/Bi_1.05_La_0.05_FeO_3_ heterostructures^22^,^23^. As a typical dielectric oxide, BaTiO_3_ has attracted extensive studies because of excellent ferroelectric and dielectric properties. For instance, high Curie temperature, positive transverse piezoelectric coefficient, and low leakage current have been obtained in (BaTiO_3_)_0.5_:(Sm_2_O_3_)_0.5_ thin films, which has been revealed to be originated from the strain at the vertical interfaces between BaTiO_3_ and Sm_2_O_3_^24^,^25^,^26^. Considering the ordered interfaces and large interfacial area, (BaTiO_3_)_1-x_:(Sm_2_O_3_)_x_ can be an unique system for investigating the relationship between the interfaces and dielectric properties. In this work, we present a comparative study on dielectric properties of (BaTiO_3_)_1-x_:(Sm_2_O_3_)_x_ nanocomposite thin films with compositions of *x* = 0.5 and 0.62. Due to a relatively large strain generated at the interfaces, vertical interfaces between BaTiO_3_ (BTO) and Sm_2_O_3_ are revealed to become the sinks to attract *V*_O_s. The movement of *V*_O_s is confined at the interfaces and hampered by misfit dislocations along the interfaces, which results to a dielectric relaxation in the (BTO)_1-x_:(Sm_2_O_3_)_x_ (BTO:Sm_2_O_3_) nanocomposite thin films.

## Results

**Figure 1:**
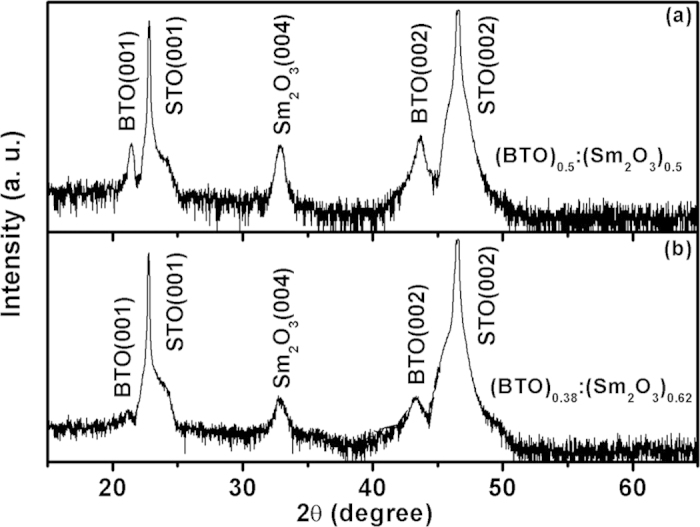
Comparison for the XRD *θ*-2*θ* scans for BTO:Sm_2_O_3_ thin films with compositions of (**a**) *x* = 0.5 and (**b**) *x* = 0.62.

Typical x-ray diffraction (*θ* – 2*θ*) patterns for the composite thin films are shown in Fig. 1. Only (00*l*) diffraction peaks appear in the patterns for both thin films and substrates, suggesting that the BTO and Sm_2_O_3_ phases coexist in the composite thin films and are preferentially oriented along the *c-*axis. According to our previous works^24^,^25^,^26^,^27^, the orientation relationship between thin films and substrates is determined to be (002)_BTO_||(002)_Sm2O3_||(002)_STO_ and [200]_BTO_||[220]_Sm2O3_||[200]_STO_. It should be noted that, due to the lattice mismatch between the BTO and Sm_2_O_3_ (the lattice constants of bulk BTO and Sm_2_O_3_ are 4.03 and 10.93 Å, respectively), misfit dislocations are thus generated for partial strain relaxation, which is confirmed by transmission electron microscopy (TEM) measurements and will be discussed later. Additionally, large residual strains of ~2.3% and ~3.4% have been found in the BTO phase in the composite thin films with compositions of *x* = 0.5 and 0.62 respectively, which is consistent with the reported results^24^,^26^.

**Figure 2:**
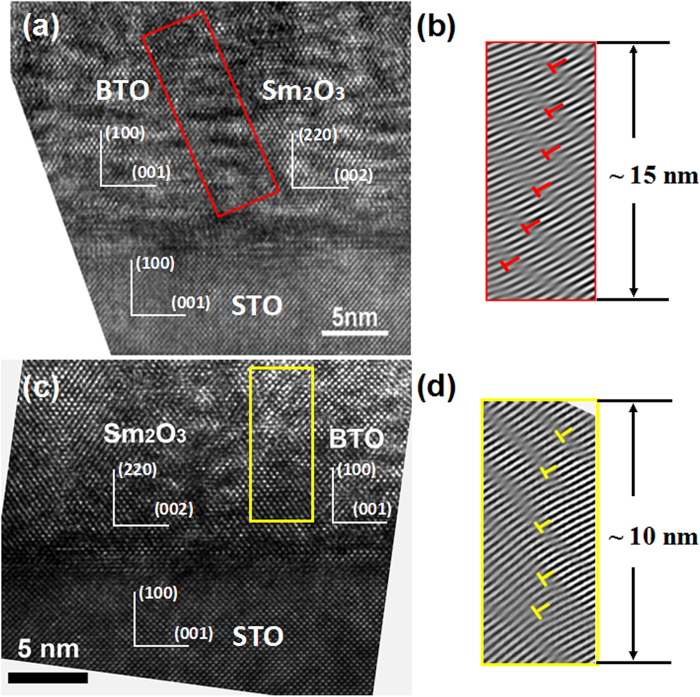
High-resolution TEM images of BTO:Sm_2_O_3_ thin films with (**a**) *x* = 0.5 and (**c**) *x* = 0.62. Corresponding Fourier-filtered (FFT) images along column boundaries are shown as (**b**) and (**d**), respectively. The FFT images are enlarged to show misfit dislocations clearly.

In previous works, we have revealed that the BTO and Sm_2_O_3_ phases grow alternatively and spontaneously and form a vertically aligned columnar structure in the BTO:Sm_2_O_3_ thin films^24^,^25^,^26^. Fig. 2(a) show high resolution TEM images of the BTO:Sm_2_O_3_ thin films with compositions of *x* = 0.5 and 0.62 respectively, which demonstrate the excellent heteroepitaxial growth of the BTO and Sm_2_O_3_ on the STO substrates. Combined with previous results^24^,^25^,^26^, these images indicate that self-assembled Sm_2_O_3_ nanocolumns are evenly sized, distributed, and embedded in a BTO matrix. And the diameter of single Sm_2_O_3_ nanocolumn is about 10 nm. So, the density of interfaces is estimated to be about 10^8^/m. More than this, a periodic arrangement of misfit dislocations is found along the vertical interfaces, as shown in the corresponding Fourier-filtered images in Fig. 2(b). The density of misfit dislocations along the interfaces is estimated to be about 4.0 × 10^8^/m for *x* = 0.5, and about 5.0 × 10^8^/m for *x* = 0.62. Considering the density of interfaces, the areal density of misfit dislocations is estimated to be about 4.0 × 10^16^/m^2^ for *x* = 0.5, and about 5.0 × 10^16^/m^2^ for *x* = 0.62. In other words, the density of misfit dislocations is very high in the BTO:Sm_2_O_3_ thin films, which may originate from the large lattice mismatch between the BTO and Sm_2_O_3_. Besides, the density of misfit dislocations for *x* = 0.5 is lower than that for *x* = 0.62. All these results suggest that self-assembled vertical heteroepitaxial nanostructures of BTO:Sm_2_O_3_ are synthesized as expected and can be used as model system to explore the relationship between the vertical interfaces and dielectric properties in oxide thin films.

**Figure 3:**
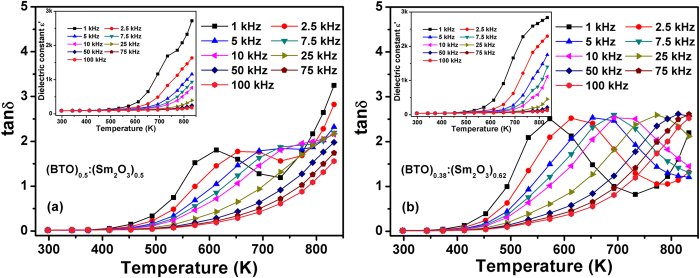
Temperature dependence of tanδ for BTO:Sm_2_O_3_ thin films with (**a**) *x* = 0.5 and (**b**) *x* = 0.62 measured at various frequencies. The insets show temperature dependence of dielectric constant.

To investigate the vertical interface effects on dielectric behavior, the temperature dependence of the real part of dielectric constant (*ε‘*) and dielectric loss (tan*δ*) are measured at the frequency ranging from 1 kHz to 1 MHz by using a structure of Pt/BTO:Sm_2_O_3_/Nb-STO (shown as Fig. 3). In general, as the frequency increases, the tan*δ* ~T curve shifts towards a higher temperature region, indicating a typical characteristic of dielectric relaxation phenomenon. Furthermore, it is obvious that *ε‘* gradually increases with increasing temperature (shown as insets of Fig. 3). It should be pointed that, because of a relatively large vertical strain observed in the BTO phase in the composite films (~2.3% and 3.4% for *x* = 0.5 and 0.62, respectively), the ferroelectric Curie temperature of the composite films may be over 833 K, which is comparable to the previous results^24^,^28^,^29^.

**Figure 4:**
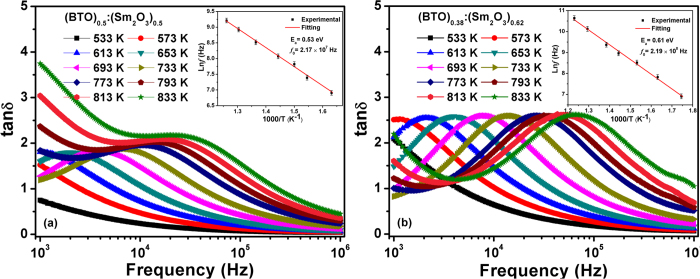
Frequency dependence of tan*δ* for BTO:Sm_2_O_3_ thin films with (**a**) *x* = 0.5 and (**b**) *x* = 0.62 measured at different temperatures. The insets show the Arrhenius plots of relaxation times. The red straight lines in insets are the linear fitting based on the Arrhenius law.

Figure 4 shows the frequency dependent tan*δ* for the BTO:Sm_2_O_3_ thin films measured at different temperatures. The peaks of tan*δ* shift towards a higher frequency region with increasing temperature, further approving the existence of dielectric relaxation in the composite thin films. In order to explore the physical mechanism of the relaxation process, we calculated the relaxation parameters for BTO:Sm_2_O_3_ thin films in terms of the Arrhenius Law


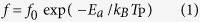


where 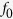 is the pre-exponential factor, 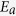 is activation energy required for relaxation process, 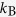 is the Boltzmann constant, and 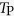 is the temperature where the maximum loss tangent occurs. The Arrhenius plots were shown as insets of Fig. 4(a). The values of 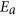 and 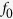 were found to be 0.53 eV and 2.17 × 10^7^ Hz for *x* = 0.5, and 0.61 eV and 2.19 × 10^8^ Hz for *x* = 0.62, respectively.

**Figure 5:**
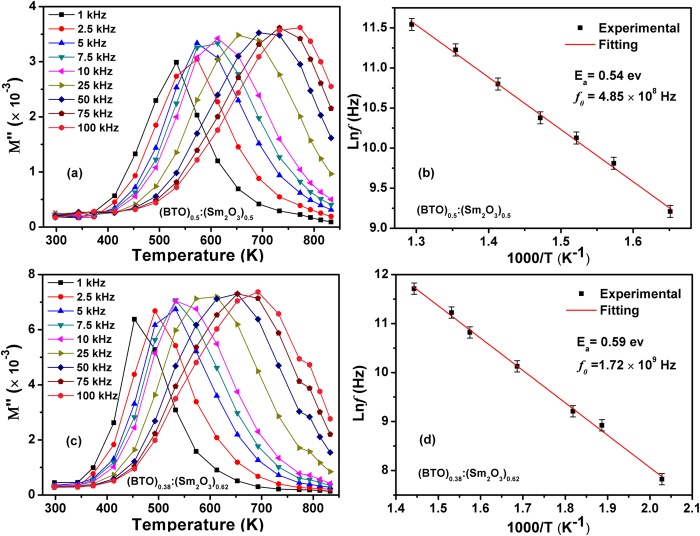
Variation of 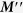 as a function of temperature for BTO:Sm_2_O_3_ thin films with (**a**) *x* = 0.5 and (**c**) *x* = 0.62 measured at different frequencies. The corresponding Arrhenius plots of the frequency against temperature were shown in (**b**) and (**d**), respectively. The solid curves are the best fits to the Arrhenius law.

To further understand the physical process of the observed dielectric relaxation in the BTO:Sm_2_O_3_ thin films, the imaginary (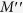) part of electric modulus (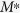) given by M "=
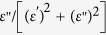
 as a function of temperature at a series of frequencies were illustrated in Fig. 5(a). As we expected, well-defined M "(T ) 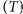 peaks have been found in the whole temperature range. The 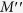
* *~* T* curve shifts towards higher temperature with increasing frequency, indicating a typical relaxation nature. The Arrhenius plots for Ln (*f*_max_) vs 10^3^/T were also shown in Fig. 5(b). Accordingly, the relaxation parameters of 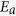 and 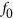 were deduced to be 0.54 eV and 4.85 × 10^8^ Hz for *x* = 0.5, and 0.59 eV and 1.72 × 10^9^ Hz for *x* = 0.62, respectively. The activation energy obtained from 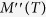 is almost the same as the calculated values from tan*δ*(*T*) (see insets of Fig. 4(a)), which further confirms that the fitting results are reasonable. It should be pointed out that, because the relaxation time (τ = 1/*f*) for 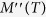 and tan*δ* (*T*) follow the general rule of τ_tan*δ*_ > τ_*M”*_^30^,^31^, the pre-exponential factor deduced for 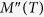 is always one order of magnitude larger than that estimated from tan*δ* (*T*).

Now it is important to investigate the origin of the dielectric relaxation in the BTO:Sm_2_O_3_ thin films. As a Pt/BTO:Sm_2_O_3_/Nb-STO vertical capacitor has been used in the dielectric measurements, the composite thin film can be reviewed as three parts connected in parallel: the BTO phase, the Sm_2_O_3_ phase, and the vertical interfaces. Up to now, as far as we know, there are no reports on dielectric relaxation in the Sm_2_O_3_. And the activation energy of BTO-based perovskite oxides is 0.88 ~ 1.56 eV^32^,^33^,^34^,^35^, which is obviously higher than those in the present work. To further exclude the influence of the BTO and Sm_2_O_3_ phases on dielectric relaxation, the dielectric properties of pure BTO and Sm_2_O_3_ thin films were measured (not shown). There is no obvious dielectric relaxation in the pure Sm_2_O_3_ thin film. And, dielectric relaxation was observed in the pure BTO film with an *E*_*a*_ value of 1.08 eV, which is in consistent with the previous results. Therefore, neither the BTO nor the Sm_2_O_3_ phase is responsible for the dielectric relaxation observed in the composite films. In other words, the vertical interfaces dominate the relaxation behavior. On the other hand, it is well known that the dielectric loss is closely correlated with the leakage current in oxide thin films. And we have demonstrated that the leakage behavior is dominated by the vertical interfaces in (BTO)_0.5_:(Sm_2_O_3_)_0.5_ thin films, which further approves that the vertical interfaces are those who resulted to the dielectric relaxation^26^. It has also been reported that the electrode interfaces related to *V*_O_s gradients affect fatigue and dielectric loss in ferroelectric oxides^36^. However, the vertical interfacial area is about twenty ~ forty times of the electrode interfacial area in the BTO:Sm_2_O_3_ thin films. Though the contribution of the electrode interfaces may dominate the dielectric behavior in the pure BTO and Sm_2_O_3_ thin films, the dielectric behavior of the BTO:Sm_2_O_3_ composite films is totally different from those of the pure thin films. And, as we discussed earlier, the density of misfit dislocations is very high in the composite thin films. Considering all these factors, the contribution of the electrode interfaces to the dielectric relaxation in the BTO:Sm_2_O_3_ thin films should be neglected in the present work.

It is well known that *V*_O_s have been demonstrated to be intrinsic defects and are often unavoidable in oxide thin films. The relaxation occurring at high temperatures are exclusively related to the *V*_O_s. And an activation energy of 0.3 ~ 1.0 eV is the typical value for relaxation behavior caused by *V*_O_s, which is verified by many previous reports^32^,^37^,^38^,^39^. According to the values obtained in the BTO:Sm_2_O_3_ thin films, the dielectric relaxation in the measured temperature region was proposed to associate with *V*_O_s. On the other hand, because of the structural discontinuity as well as the strain, the interfaces have been approved to attract and gather the *V*_O_s^6^,^40^,^41^,^42^,^43^,^44^. For example, strain-driven accumulation of *V*_*O*_s along the vertical interfaces has been observed in (REBa_2_Cu_3_O_7-δ_)_1-x_:(BaZrO_3_)_x_ composite thin films^45^. More than this, in our previous work, electron energy loss spectroscopy (EELS) measurements revealed that a large concentration of *V*_O_s forms at the vertical interfaces in (SrTiO_3_)_0.5_:(Sm_2_O_3_)_0.5_ composite thin films due to a large lattice misfit^46^. Considering a large vertical strain generated at the interfaces in the present work, the vertical interfaces are believed to become the sinks to attract *V*_O_s, which is the origin of the dielectric relaxation in the composite thin films^26^,^47^,^48^,^49^.

**Figure 6:**
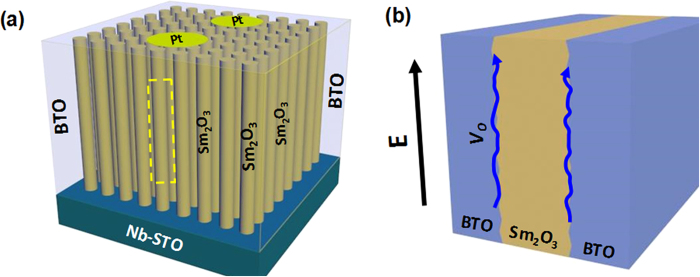
(**a**) Schematic diagram of Pt/BTO:Sm_2_O_3_/Nb-STO vertical sandwich capacitors. (**b**) The expanded view of the dashed part in the schematic diagram to show the interfaces between Sm_2_O_3_ nanocolumns and BTO matrix. E and blue lines represent the electric field and the pathway of movement of *V*_O_s, respectively.

To understand the mechanism of the observed relaxation behavior, a model has been proposed and shown in Fig. 6. In this system, as shown in Fig. 6(a), Sm_2_O_3_ are nanocolumns embedded in a BTO matrix and vertical sandwich capacitors with a configuration of Pt/BTO:Sm_2_O_3_/Nb-STO have been used to investigate the dielectric properties. Electric field is applied parallel to the interfaces between Sm_2_O_3_ and BTO (shown as Fig. 6(b)). On the other hand, *V*_O_s have been attracted at the interfaces and can be viewed as ions with positive charges^50^,^51^. With the assistance of an electric field, *V*_O_s can move along the vertical interfaces in the direction of electric field. However, the long range movement of *V*_O_s will be hampered by the misfit dislocations observed in the vertical interfaces, which results to the dielectric relaxation of the BTO:Sm_2_O_3_ thin films. In addition, with the increasing density of misfit dislocations, the values of the activation energy varied from 0.53 to 0.61 eV, further confirming the above mechanism.

The outcome of our above analysis shows that self-assembled vertically aligned nanocomposite thin films have three unique features: ordered vertical interfaces, large interfacial area, and *V*_O_s gathered at the vertical interfaces^26^,^45^,^46^,^47^,^48^,^49^. With the assistance of an electric field, *V*_O_s can only move up and down along the vertical interfaces, which means that the transportation of *V*_O_s has been effectively confined. Meanwhile, misfit dislocations formed along the vertical interfaces can be used to manipulate the dynamics of *V*_O_s. These unique features are very helpful to investigate the transportation mechanism of *V*_O_s, and to enhance ion conductivity in oxides, which play a key role in determining the performance of energy conversion and storage devices, such as thin films solid oxide fuel cells, photocatalysts, and batteries^52^,^53^,^54^.

## Discussion

In summary, epitaxial (BTO)_1-x_:(Sm_2_O_3_)_x_ vertically aligned nanocomposite thin films with compositions of *x* = 0.5 and 0.62 have been fabricated by pulsed laser deposition, which were used as model system to investigate the relationship between the microstructure, the interfaces, and the dielectric behavior. The structural discontinuity and a relatively large residual strain attract the accumulation of *V*_O_s at the vertical interfaces between the BTO and Sm_2_O_3_. With the assistance of an electric field, the movement of *V*_O_s has been confined along the interfaces and been hampered by the misfit dislocations, which results to an interface-induced relaxation behavior. The present work has broad implications for the understanding of the correlation between the interfaces and physical properties, for the manipulating or optimizing of functionalities in the nanocomposite oxide thin films, and for the utilization of dielectric materials in high-temperature applications. More than this, the unique characteristics of vertically aligned nanocompsite thin films present potential applications in energy conversion and storage devices.

## Methods

Epitaxial (BTO)_1-x_:(Sm_2_O_3_)_x_ thin films with compositions of *x* = 0.5 and 0.62 were deposited on (001) oriented SrTiO_3_ (STO) and Nb-doped SrTiO_3_ (Nb-STO) substrates by pulsed laser deposition (PLD) with a KrF excimer laser (Lambda Physik, λ = 248 nm). A laser fluence of ~2 J/cm^2^ with a repetition rate of 3 Hz were focused onto composite targets with different molar ratios. An optimized substrate temperature of 720 ^o^C and oxygen pressure of 25 Pa were used during depositions. Immediately following depositions, films were annealed *in situ* for one hour at a temperature of 450 ^o^C and an oxygen pressure of 0.8 atm. X-ray diffraction (XRD, Rigaku K/Max) and transmission electron microscopy (TEM, FEI Tecnai F20 analytical microscope) were used to investigate the microstructure of thin films. The thickness of thin films was measured by cross-sectional TEM.

For electrical measurements, vertical sandwich capacitors with a configuration of Pt/BTO:Sm_2_O_3_/Nb-STO were fabricated, where thin films with a thickness of ~200 nm were used and Pt top electrodes with an area of 8 × 10^−4^ cm^2^ were deposited by sputtering. The dielectric properties were investigated using an Agilent 4294 A Impedance Analyzer. The measurements were performed at selected temperatures in a Linkam Scientific Instruments HFS600E-PB4 system.

## Additional Information

**How to cite this article**: Li, W. *et al.* Vertical Interface Induced Dielectric Relaxation in Nanocomposite (BaTiO_3_)_1-x:_(Sm_2_O_3_)_x_ Thin Films. *Sci. Rep.* **5**, 11335; doi: 10.1038/srep11335 (2015).
